# Supplementary material for: Splice-Junction-Based Mapping of Alternative Isoforms in the Human Proteome
Source: Cell Rep. Author manuscript; Available in PMC 2020 Jan 15. (PMC6961840; doi:10.1016/j.celrep.2019.11.026)
Supplement: 2 [file NIHMS1546469-supplement-2.zip › DF1/PXD010154/Esophagus/Esophagus_8_SVIL_DSEGDTPSLINWPSSK.pdf]

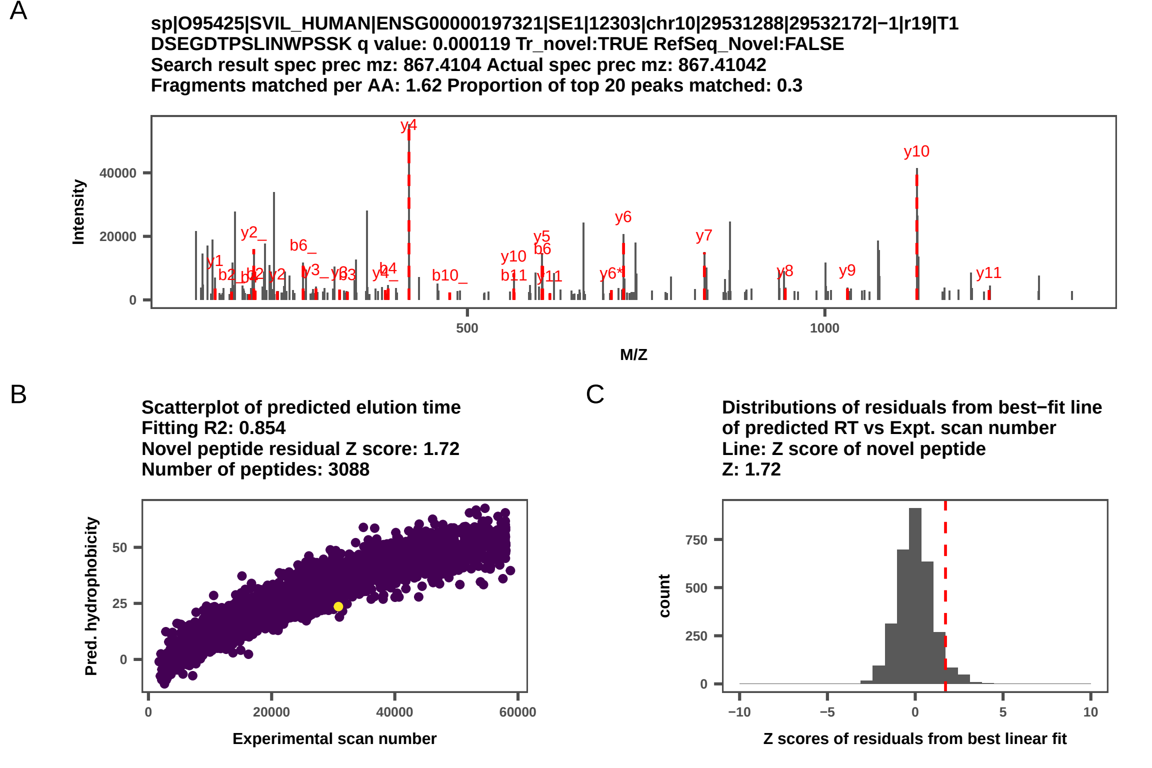
A
sp|O95425|SVIL_HUMAN|ENSG00000197321|SE1|12303|chr10|29531288|29532172|−1|r19|T1
DSEGDTPSLINWPSSK q value: 0.000119 Tr_novel:TRUE RefSeq_Novel:FALSE
Search result spec prec mz: 867.4104 Actual spec prec mz: 867.41042
Fragments matched per AA: 1.62 Proportion of top 20 peaks matched: 0.3
Intensity
40000
20000
0
M/Z
500
1000
y4
y10
y6
y7
y5
b6
y10
b11
y11
y6*
y8
y9
y11
b10_
b6_
y2_
y1
b4
y4_
B
Scatterplot of predicted elution time
Fitting R2: 0.854
Novel peptide residual Z score: 1.72
Number of peptides: 3088
Pred. hydrophobicity
50
25
0
0
20000
40000
60000
Experimental scan number
C
Distributions of residuals from best−fit line
of predicted RT vs Expt. scan number
Line: Z score of novel peptide
Z: 1.72
count
750
500
250
0
−10
−5
0
5
10
Z scores of residuals from best linear fit
